# Epistasis between Beneficial Mutations and the Phenotype-to-Fitness Map for a ssDNA Virus

**DOI:** 10.1371/journal.pgen.1002075

**Published:** 2011-06-02

**Authors:** Darin R. Rokyta, Paul Joyce, S. Brian Caudle, Craig Miller, Craig J. Beisel, Holly A. Wichman

**Affiliations:** 1 Department of Biological Science, Florida State University, Tallahassee, Florida, United States of America; 2 Department of Mathematics and Statistics, University of Idaho, Moscow, Idaho, United States of America; 3 Department of Biological Sciences, University of Idaho, Moscow, Idaho, United States of America; Fred Hutchinson Cancer Research Center, United States of America

## Abstract

Epistatic interactions between genes and individual mutations are major determinants of the evolutionary properties of genetic systems and have therefore been well documented, but few quantitative data exist on epistatic interactions between beneficial mutations, presumably because such mutations are so much rarer than deleterious ones. We explored epistasis for beneficial mutations by constructing genotypes with pairs of mutations that had been previously identified as beneficial to the ssDNA bacteriophage ID11 and by measuring the effects of these mutations alone and in combination. We constructed 18 of the 36 possible double mutants for the nine available beneficial mutations. We found that epistatic interactions between beneficial mutations were all antagonistic—the effects of the double mutations were less than the sums of the effects of their component single mutations. We found a number of cases of decompensatory interactions, an extreme form of antagonistic epistasis in which the second mutation is actually deleterious in the presence of the first. In the vast majority of cases, recombination uniting two beneficial mutations into the same genome would not be favored by selection, as the recombinant could not outcompete its constituent single mutations. In an attempt to understand these results, we developed a simple model in which the phenotypic effects of mutations are completely additive and epistatic interactions arise as a result of the form of the phenotype-to-fitness mapping. We found that a model with an intermediate phenotypic optimum and additive phenotypic effects provided a good explanation for our data and the observed patterns of epistatic interactions.

## Author Summary

Epistasis, the extent to which the effects of a mutation depend on its genetic context, can have profound effects on the evolutionary process and strongly affects our understanding of the prevalence of sexual reproduction. It has been investigated in a diverse array of organisms but almost exclusively for deleterious mutations. Interactions between beneficial mutations can impede adaptation, and we therefore investigated epistasis between beneficial mutations by constructing 18 bacteriophage genomes, each with two mutations that had been previously identified as beneficial, and measuring their fitnesses. We found universal evidence for epistasis—every pair of mutations conferred fitness lower than that expected from the single mutations alone. In many cases, a beneficial mutation became deleterious when in combination with another, and in fact, only one pair out of 18 could be shown to confer significantly greater fitness than its constituent mutations alone. To explain these results, we developed a model of the relationship between phenotype and fitness that posits an intermediate phenotypic optimum and assumes no epistasis at the phenotypic level. This model fit our data well and showed that the patterns we observed could result because mutants have phenotypes that overshoot the optimum.

## Introduction

The nature of epistatic interactions between loci or mutations is a major component of evolutionary theories. For example, epistasis is thought to have been important in the evolution of sexual reproduction [1], [2] and reproductive isolation between incipient species [3]–[6]. In models of adaptation and fitness landscapes, epistatic interactions are the primary determinant of the topology of landscape and thus the accessibility of high-fitness genotypes [7]–[11]. Previous empirical studies have provided much evidence for a variety of forms of epistasis. Compensatory mutations, whose beneficial effects depend on the presence of a deleterious mutation, provide direct evidence of the relevance of epistasis; numerous empirical examples have been described [12]–[17]. Experiments in microbial [18], [19] and viral systems [20]–[24] have provided abundant evidence for antagonistic epistasis, in which the total effect of multiple mutations is less than expected on the basis of their individual effects. Similarly, some of these same studies have provided evidence for synergistic epistasis [18], [22], [23], in which the combined effects of mutations are greater than expected. Some evidence suggests that the predominance of antagonistic epistasis is a feature of simpler genomes, whereas synergistic epistasis is more common in more complex genomes [25].

The majority of commonly cited effects of epistasis in evolution are the results of interactions between deleterious alleles, but interactions between beneficial alleles can significantly affect the rate of adaptation. Epistasis has been shown to constrain pathways of molecular adaptation severely [26]–[28]. One of the major advantages of sexual reproduction is the presumed benefit of recombining separate beneficial mutations or alleles into the same genome [2]. Discussions of microbial evolution are dominated by the phenomenon of clonal interference [29]–[37], in which, because of their asexual mode of reproduction, clonal organisms suffer a reduced rate of adaptation because individual beneficial mutations must compete for fixation rather than being combined into the same genome by recombination. These results rest on the assumption that mutations that are individually beneficial remain beneficial when combined. Furthermore, many models of adaptation rely on the assumption that the effects of beneficial mutations are additive [29], [30], [38]. Though these assumptions are widely used, their validity is largely undetermined.

To explore epistatic interactions between beneficial mutations, we constructed bacteriophage mutants with pairs of previously identified beneficial mutations by site-directed mutagenesis. We used nine beneficial mutations (which we designate A through I, in order of their appearance in the genome; Table 1) identified for the ssDNA microvirid bacteriophage ID11 [39]. This phage infects *Escherichia coli* strain C, and the nine mutations increased growth rate of the wild type at 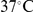 in liquid culture with excess hosts. We built 18 of the possible 36 pairs of these nine beneficial mutations (designated by two-letter combinations) and measured the fitnesses of the wild-type genotype, those of the single beneficial mutations, and the double mutants. A similar approach was used to study epistatic interactions between deleterious mutations and between beneficial mutations for the RNA virus vesicular stomatitis virus (VSV) [22], but we go beyond characterizing the patterns by constructing an explanatory model that posits that epistatic interactions arise at the level of the mapping from phenotypes to fitness and assessing the fit of our data to it.

**Table 1:** Nine mutations beneficial to the ssDNA bacteriophage ID11.

Label	Protein function	Protein name	Aa position	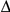 Aa	Nuc position	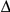 Nuc
A	DNA binding	J	15	A 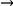 V	2520	C 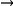 T
B	DNA binding	J	20	V 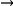 L	2534	G 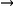 T
C	coat	F	3	V 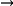 F	2609	G 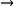 T
D	coat	F	314	A 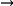 V	3543	C 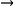 T
E	coat	F	322	N 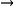 S	3567	A 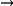 G
F	coat	F	355	P 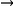 S	3665	C 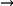 T
G	coat	F	416	M 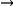 I	3850	G 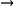 A
H	coat	F	419	T 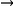 A	3857	A 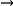 G
I	coat	F	421	D 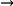 G	3864	A 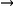 G

The nine beneficial mutations used in this study affect two different viral proteins: the DNA binding protein J and the major coat protein F. Positions are based on the published genome sequence of ID11 (GenBank accession # AY751298). Nuc, nucleotide; 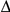
Nuc, nucleotide change.

## Results/Discussion

### Antagonistic epistasis between beneficial mutations

**Figure 1:**
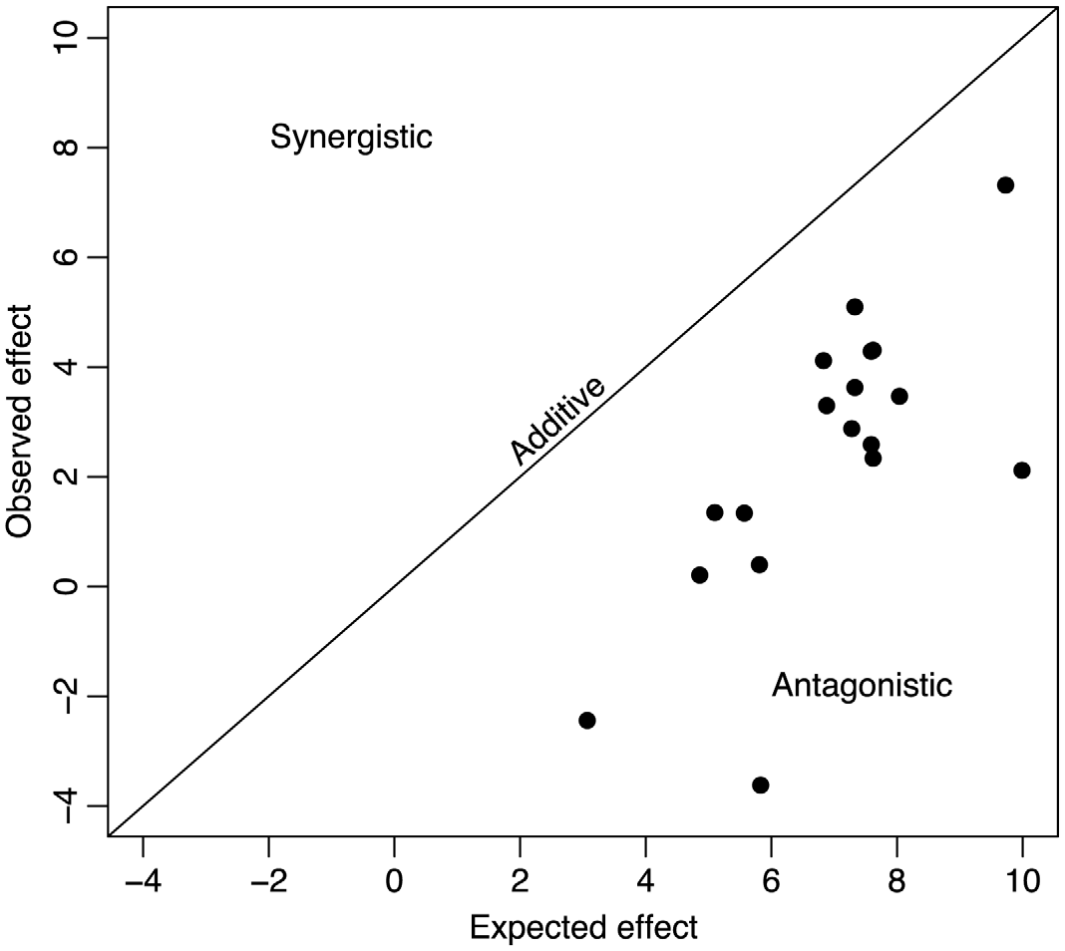
Universal antagonistic epistasis for beneficial mutations. The fitness of double mutant ID11 phage expected on the basis of addition of the effects of the two mutations is plotted against the observed effects on the doubles mutants. Additive effects would fall on the diagonal, synergistic effects would fall above the diagonal, and antagonistic effects would fall below the diagonal. Effects are given in units of doublings per hour.

For the 18 double mutants, the expected effect of incorporating both beneficial mutations into the genome under additivity (i.e., without epistasis) was greater than the observed effect (Figure 1). Because our fitness was measured as a growth rate (i.e., log fitness), the expectation under additivity was that the effect of the two mutations in combination would be the sum of the single-mutant effects on growth rate. We can measure the deviation from additivity by calculating
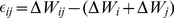
(1)where 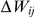 is the effect of the double mutant with mutations 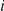 and 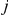 relative to the wild type, and 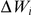 is the effect of single mutant 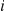 relative to the wild-type. An 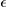 of 0 implies additivity; 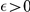 implies synergistic epistasis, and 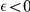 implies antagonistic epistasis [22]. The average deviation from additivity over the 18 double mutants was 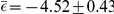. We could easily reject additivity (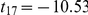, 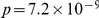). All deviations were less than zero (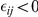 for all 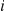 and 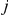), and the deviation of smallest magnitude, 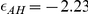, was more than 5 standard errors less than zero. We therefore found no evidence of synergistic epistasis between beneficial mutations and could strongly reject additivity. Epistasis between beneficial mutations of ID11 was entirely antagonistic. Previous work with the RNA virus VSV looking at the effects of pairs of beneficial mutations also found evidence for a predominance of antagonistic epistasis and no significant cases of synergistic epistasis for beneficial mutations. This result confirmed the prediction by Martin et al. [40] based on a generalized version of Fisher's geometrical model [41] that values of 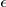 between pairs of beneficial mutations should be skewed toward negative values (see below for a full treatment of this model).

### Decompensatory epistasis for beneficial mutations

Although, under antagonistic epistasis, the beneficial effect of a second mutation is reduced, that second mutation might still increase fitness to some lesser extent. We are also therefore interested in decompensatory epistasis [22], under which a beneficial mutation actually becomes deleterious in the presence of another beneficial mutation (analogous to compensatory mutations, which are beneficial only in the context of a deleterious mutation). Decompensatory epistasis is also a special case of sign epistasis [9] and would indicate that the set of beneficial mutations available for the wild-type genotype may be quite different from the set of beneficial mutations available after the first fixation event in adaptation. This situation would be consistent with, for example, the standard implementation of the mutational landscape model [42]–[45], which uses a random fitness landscape. After a mutation becomes fixed in the population, an entirely new set of beneficial mutations (if any) becomes available to the evolving population.

**Figure 2:**
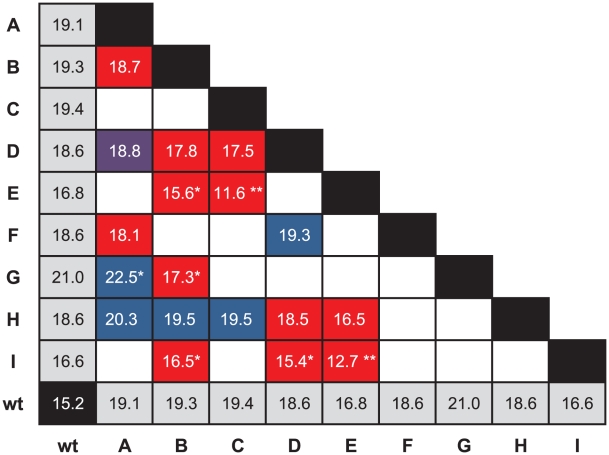
Evidence for decompensatory epistasis. The grid shows the fitnesses of the wild type, single mutants, and double mutants. Empty cells represent the double mutants that were not constructed. Red indicates that the average fitness of the double mutant is lower than the average fitness conferred by its two constituent single mutations. Blue indicates that its fitness is higher than that of either single mutant, and purple indicates that it is between the fitnesses of the two single mutants. A “*” in a red box indicates the double mutation confers a fitness significantly lower than that conferred by one single mutation, and a “**” indicates that the double mutation confers a fitness significantly lower than that conferred by either of its single mutations. A “*” in a blue box indicates that the double mutation confers a fitness significantly higher than that conferred by either constituent single mutation.

Figure 2 illustrates the cases in which the mean fitness conferred by the double mutation is less than the mean fitness conferred by one or both beneficial mutations on their own. To test for significance, we performed three different sets of tests of increasing stringency. For the first, we simply asked whether the fitness conferred by the double mutation was significantly less than the higher of the two fitnesses conferred by the single mutations of which it was composed. We called the situation in which it was conditional decompensatory epistasis, as it merely guaranteed that at least one of the two mutations was deleterious in the presence of the other and did not preclude the case where the double-mutant fitness lies between the two single-mutant fitnesses. Using a one-sided Welch two-sample *t*-test and a Bonferroni correction for 18 tests, we found six double mutants that showed evidence of conditional decompensatory epistasis with 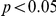: BE, BG, BI, CE, DI, and EI. The second test was to determine whether the double mutant was less fit that the lower-fitness single mutant. We refer to the case in which it was as unconditionally decompensatory epistasis, as regardless of the order mutations might be added to the genome, the second was always deleterious. Using the same test as above, we found only two double mutants that were unconditionally decompensatory with 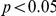: CE and EI. Finally, our most stringent test was to ask whether the double mutant was less fit than the wild-type genotype. This situation would imply that the two mutations together constituted a deleterious mutation, i.e., a population in which both mutations became fixed would be worse off than one in which neither had. Using the same test as above, we found two double mutants that were significantly less fit than the wild type with 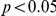: CE and EI, the two unconditionally decompensatory doubles.

The presence of decompensatory epistasis for beneficial mutations is consistent with a random fitness landscape, but clearly not all pairs of beneficial mutations show this pattern. In fact, at least one double mutant is significantly more fit than mutants bearing either of its constituent single mutations (see below). Nevertheless, in a number of cases, both beneficial mutations could not become fixed in the population because they could not outcompete one or both of the single mutations from which they were formed. A similar observation about beneficial mutations was made for VSV [22]. Under landscape models such as the block model [10], [11] or 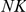 model [7], [8], the ruggedness of the landscape can be adjusted if the extent of epistatic interactions is changed from a smooth, additive landscape with no epistasis to a highly rugged, highly epistatic random landscape. We can clearly reject the nonepistatic model, but just as clearly, the random landscape is too extreme. Under a random-landscape model, the probability that a second mutation increases fitness (i.e., is not decompensatory) is the same as the probability that a random mutation is beneficial, which is generally assumed to be small. Our observation of nondecompensatory mutations is therefore inconsistent with this model.

### The advantage of sex

One of the major proposed advantages of sexual reproduction is that it facilitates recombination, which can increase the rate of adaptation by allowing beneficial mutations arising in different genomes to be combined in the same genome. This advantage is contigent on the assumption of a fitness increase for the recombinant over its composite single mutations. To test this assumption, we asked whether any of the 18 double mutants had significantly higher fitness than the higher of the fitnesses of mutants bearing the single mutations of which it was composed. Using a one-sided Welch two-sample *t*-test and Bonferroni correction for 18 tests, we found only a single double mutation that could outcompete its constituent single mutations: AG (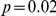 with Bonferroni correction). Even without the Bonferroni correction, only two doubles are significantly higher at the 5% significance level: AG (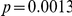) and AH (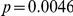). Therefore, recombination would not increase the rate of adaptation in this phage system.

This observation, together with the presence of decompensatory epistasis described above, indicates that the patterns predicted by clonal interference models [29], [30] may actually arise even in the presence of recombination. The assumption of the model is that, because of their asexual mode of reproduction, clonal organisms have a lower rate of adaptation because individual beneficial mutations must compete with one another for fixation rather than be combined into the same genome through recombination for simultaneous fixation. If combinations of beneficial mutations confer less fitness or not more fitness than the single mutations, however, even with recombination, the single mutations must compete for fixation because of a kind of epistatic interference or epistatic repulsion. Our results suggest that the types of theoretical results derived for asexuals have broader applicability even in sexual organisms, while at the same time calling into question the underlying impetus for the models, if similar results are found in other systems. In other words, in our phage, sexual reproduction would provide little or no increase in the rate of adaptation, because ultimately one of the single mutants will outcompete the other singles and any double mutants that could be produced by recombination.

### Additivity of phenotypic effects

**Figure 3:**
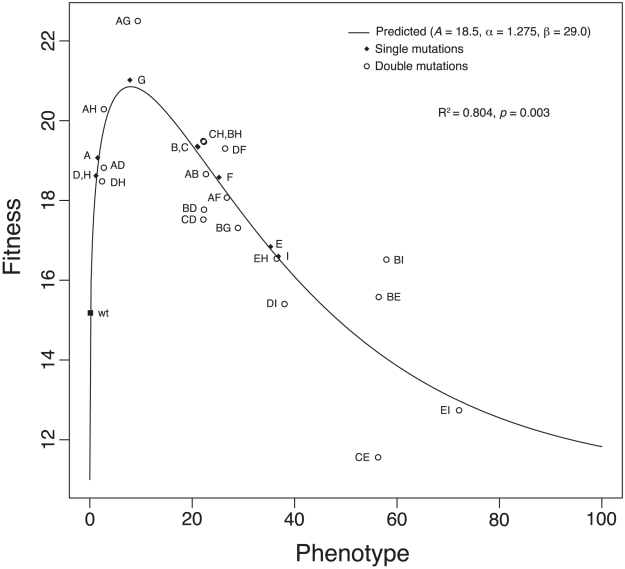
The phenotype-to-fitness map. The plot shows the fit of our model for the phenotype-to-fitness map. The model assumes a gamma curve for the relationship between fitness and phenotype. Phenotypic effects were assumed to be additive and epistasis for fitness to arise through the shape of the curve. The variance of the normal error was estimated to be 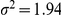. 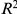 gives the coefficient of determination. The 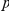 value is based on an 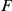 test comparing our model to a model assuming that single- and double-mutant fitnesses are independent of each other. For these data, 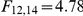. We rescaled fitness by substracting 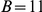 rather than the fitness of the wild type to avoid negative values.

Clearly, our results and Figures 1 and 2 reveal significant epistatic interactions between the nine beneficial mutations in our data set. Recent theoretical and empirical work has suggested that mutations produce additive biochemical effects [26], [46], and bacteriophage growth is merely a somewhat complex biochemical reaction. If phenotypic (e.g., biochemical) effects are completely additive, epistatic interactions might still arise through nonlinearity in the mapping from phenotype to fitness [40]. In addition, work with the nine beneficial mutations we studied revealed a distinct upper bound on fitness effects for beneficial mutations [47]. Such an upper bound could arise naturally with an intermediate phenotypic optimum (i.e., stabilizing selection). To determine whether such a scenario might apply to the ID11 system, we developed a simple model of the phenotype-to-fitness mapping and fit it to our data. Our model is analogous in structure to the model of Martin et al. [40], who assumed a fitness landscape based on Fisher's geometrical model [41] in a multidimensional phenotype space, additivity of phenotypic effects of mutations, and a Gaussian fitness function to map phenotypes to fitness (see below for a comparison of the two models). DePristo et al. [46] also assumed additivity of phenotypes in their model. For our model, we assumed the phenotype-fitness relationship took the form of a gamma curve, with shape (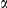), scale (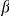), height (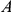), and shift (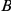) parameters. We also assumed that the mutations were all affecting a single underlying and unknown phenotype. Under the model, we assumed that the phenotype of the double mutant with single mutations 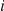 and 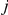 with phenotypes 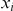 and 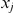 was given by 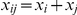. We treated the phenotypes of the single mutations as missing data and imputed their values and estimated the values of the gamma parameters 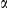, 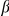, 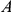, and 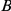. For our nine single mutants and the 18 constructed double mutants, we found that the model provides a good fit to our data (Figure 3), with a coefficient of determination 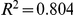. We rejected a null model that assumed the fitnesses of the doubles and the singles to be independent draws from a normal probability distribution with 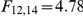 giving 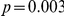. The parameter estimates for the phenotype-to-fitness map were 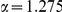, 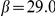, 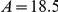, and 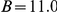. This distribution is right skewed and suggested that our wild-type ID11 is close to the phenotypic optimum.

**Figure 4:**
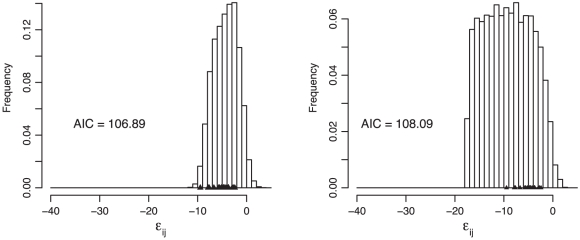
Comparison between the gamma model (left) and the model of Martin et al. [**40**] (right). The plots show the predicted distributions of the deviations from additivity (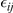; Equation 1) based on simulations under the two models. The observed values are plotted as triangles (Table 3). The gamma model fits the data better by approximately 12 log likelihoods but requires the estimation of 12 more parameters. The Akaike Information Criterion (AIC) scores of the two models are therefore similar, indicating that the two explain the data equally well.

Our gamma model and the model of Martin et al. [40] make similar assumptions but differ in the number of phenotypic dimensions and the shape of the phenotype-fitness map. Martin et al. assume a Gaussian map. To compare the performance of the models, we produced predicted distributions of epistatic effects (Figure 4). The gamma model provided a 12 log-likelihood improvement over the model of Martin et al. but requires imputation of nine phenotypes and estimates of five parameters (four for the gamma and one for the error distribution). The model of Martin et al. has only two parameters, leaving a difference of 12 parameters. Therefore, when Akaike Information Criterion (AIC) scores were used to penalize for over-fit, the two models explained the data equally well (Figure 4). Both models predicted a pattern of negative epistatic effects, which was reflected in the data, but the model of Martin et al. predicted more extreme antagonistic epistasis than was observed. The lack of fit for this model is due primarily to this prediction of extreme negative epistasis. The pattern of epistasis predicted by the gamma model is consistent with the data, but this model is penalized for extra parameters.

The gamma model assumes that the phenotypic optimum is intermediate, and our fitted values suggested that five of the nine single mutants actually overshoot this optimum. Therefore, adding two of these effects together had an overall tendency to reduce fitness, except for those mutations conferring the smallest phenotypic effects, A, D, and H (Figure 3). Note that all cases in which the second mutation appeared to have increased fitness involved at least one of these three mutations (Figure 2). In addition, the strongest epistatic interactions (i.e., those involving the unconditionally decompensatory mutations) involved at least one of the mutations with the largest phenotypic effects, E and I (Figure 3). Therefore, the model did explain the major patterns in our data, and it also made a number of testable predictions. For example, we can predict which of the 18 unconstructed possible double mutants will have low or high fitness or predict the fitness of triple mutants and beyond. To test the predictive power of the model, we conducted a series of analyses, each of which involved the removal of one of the 18 double mutants from the data set. The model was fit to each reduced data set, then used to predict the removed value. The model generated accurate predictions for 17 of the 18 double mutants (Table 2), suggesting good predictive power. More interestingly, the model predicts that, if we can change the phenotypic optimum by, for example, changing the environment, we can entirely alter the patterns of epistasis. Increasing the distance of the wild type from the optimum might produce additive effects or even synergistic epistasis rather than the uniform antagonistic effects we observed. Intriguingly, recent work on the phage 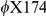, a close relative of our phage ID11, showed that epistatic interactions between different amino-acid residues at two particular sites in the phage coat protein can change from antagonistic to synergistic depending on the environment in which fitness is measured [23]. Our simple model can evince such behavior in response to simple changes in the optimum.

**Table 2:** The predictive power of the gamma model.

Removed	Predicted	Observed	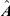	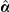	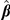	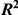	# SD
AB	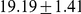	18.66	14	1.06	23	0.81	0.37
AD	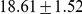	18.82	13	1.10	20	0.78	0.14
AF	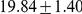	18.07	14	1.08	40	0.81	1.26
AG	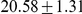	22.50	14	1.11	48	0.80	1.46
AH	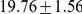	20.29	15	1.20	27	0.75	0.34
BD	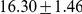	17.77	15	1.17	49	0.80	1.01
BE	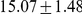	15.58	15	1.15	21	0.78	0.34
BG	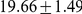	17.31	15	1.21	42	0.78	1.58
BH	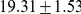	19.47	15	1.18	48	0.77	0.11
BI	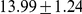	16.52	15	1.06	30	0.85	2.05
CD	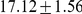	17.52	12	1.08	20	0.76	0.26
CE	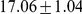	11.56	13	1.05	59	0.85	5.30*
CH	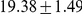	19.49	15	1.16	29	0.78	0.08
DF	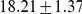	19.30	15	1.11	22	0.82	0.79
DH	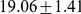	18.48	15	1.08	38	0.81	0.41
DI	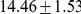	15.40	15	1.19	20	0.76	0.62
EH	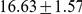	16.54	15	1.20	27	0.76	0.06
EI	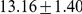	12.74	14	1.06	31	0.77	0.30

Each analysis consisted of removing one of the 18 double mutants, fitting the model to the remaining data, and predicting the fitness of the removed double mutant. For simplicity, we assumed the same shift (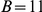) for each analysis. The last column gives the magnitude of the difference between the observed and predicted values as a number of standard deviations. A “*” indicates a significant difference at a 5% significance level from the model predictions with 15 degrees of freedom. A difference greater than 2.13 standard deviations is significant.

## Materials and Methods

### Constructing the mutants and fitness assays

The isolation and initial characterization of the nine beneficial mutations of the microvirid bacteriophage ID11 [48] have been described in detail previously [39], [48]. These mutations confer an increased growth rate on the wild-type ID11. The isolates used were confirmed by full-genome sequencing to have the mutations of interest and no other mutations.

PCR-based construction of the double mutants was based on published techniques [23], [49]. Pairs were selected such that each mutation was found in multiple genotypes, and all combinations of large-, intermediate-, and small-effect mutations were included. To construct the double mutants, we added the second mutation into a sequence-confirmed isolate of the first. We PCR amplified the circular genome in two halves, in which the forward primer for one half and the reverse of the other had the mutation to be incorporated. The other primers were selected to result in an overlap of the resulting genome halves. These halves were cleaned with a Qiagen QIAquick PCR purification kit and combined in a PCR (no primers). This reaction was cleaned with the QIAquick kit and electroporated into *E. coli*. The resulting plaques were picked and plaque purified by replating. We then subjected the final isolate to full-genome sequencing to confirm the incorporation of the mutation and the lack of secondary mutations.

Fitness assays were performed as described previously [12]. We measured fitness as the 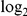 increase in the phage population per hour on *E. coli* strain C at 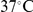. Assays were performed in an orbital water bath shaking at 200 rpm. We measured each genotype at least five times (Table 3).

**Table 3:** Fitnesses and fitness effects of all genotypes tested.

Genotype	Fitness	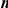	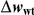	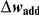	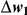	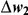	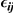
ID11	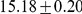	14	-	-	-	-	-
A	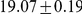	5	3.89	-	-	-	-
B	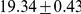	5	4.15	-	-	-	-
C	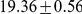	6	4.18	-	-	-	-
D	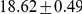	7	3.44	-	-	-	-
E	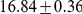	5	1.65	-	-	-	-
F	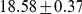	5	3.39	-	-	-	-
G	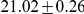	6	5.84	-	-	-	-
H	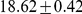	5	3.44	-	-	-	-
I	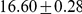	5	1.42	-	-	-	-
AB	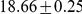	5	3.47	8.04	−0.42	−0.68	−4.57
AD	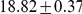	5	3.63	7.33	−0.26	0.19	−3.70
AF	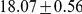	5	2.88	7.28	−1.00	−0.51	−4.40
AG	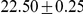	5	7.32	9.73	3.43	1.48	−2.41
AH	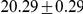	5	5.10	7.33	1.21	1.66	−2.23
BD	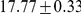	5	2.59	7.59	−1.56	−0.85	−5.00
BE	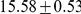	6	0.40	5.8	−3.75	−1.26	−5.40
BG	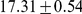	7	2.12	9.99	−2.03	−3.72	−7.87
BH	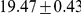	5	4.29	7.59	0.14	0.85	−3.30
BI	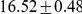	7	1.34	5.57	−2.82	−0.08	−4.23
CD	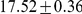	6	2.34	7.62	−1.84	−1.10	−5.28
CE	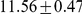	6	−3.62	5.83	−7.80	−5.28	−9.45
CH	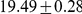	5	4.31	7.62	0.13	0.87	−3.31
DF	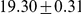	5	4.12	6.83	0.68	0.72	−2.71
DH	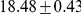	5	3.30	6.88	−0.14	−0.14	−3.58
DI	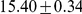	5	0.21	4.86	−3.23	−1.20	−4.65
EH	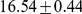	5	1.35	5.09	−0.30	−2.09	−3.74
EI	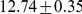	5	−2.44	3.07	−4.09	−3.86	−5.51

Fitnesses are given as the average plus or minus the standard error. The column labeled 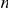 gives the number of replicate assays for each genotype. The fitness effect relative to the wild type is designated by 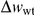. 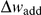 gives the fitness effect expected on the assumption that the effects of single mutations were additive, 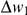 gives the effect of adding the first mutation in the genotype name into the background of the second, and 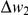 gives the effect of adding the second mutation in the genotype name into the background of the first.

### Testing for additivity of phenotypic effects

Let 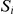 be the fitness effect of mutation 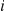 and let 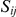 be the fitness effect of the double mutant with mutations 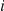 and 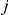. We assumed the phenotype-to-fitness mapping followed a gamma curve given by
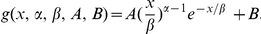
Note that this is not a probability density function. We view 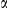 as the shape parameter, 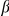 as the scale parameter, 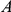 as the height parameter, and 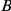 as the shift parameter. The phenotypic effect is denoted by 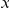. Our model is then given by 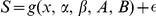 where 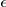 is normally distributed with mean zero and variance 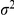. Our data consisted of the fitness effects of single mutations, 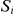, and fitness effects of double mutants, 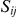; average effects are given by 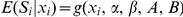 and additivity of phenotypic effects was modeled on the assumption that 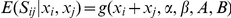.

For model fitting, the estimate of the shift parameter 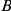, denoted by 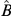, was based on the fitness of the lowest-fitness genotype (see below). We treated 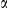, 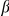, and 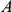 as parameters and the phenotypes 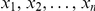 as missing data. We first imputed the phenotypes and estimated the parameters from nonlinear least squares regression. Let the array of phenotypes be 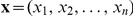. We then minimize
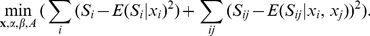
(2)We denote the estimates and imputations by 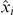, 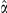, 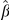, 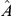, and 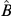. Then the predicted fitness are 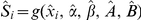 and 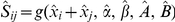.

To assess model fit, we used a simple null model where 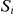 and 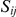 are draws from some probability distribution and vary about some mean 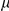 such that 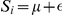 and 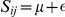, where 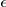 follows a normal distribution with mean zero and variance 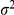. Under this null model, the fitnesses of the single mutations and double mutations are completely independent of one another. We can therefore consider 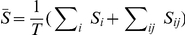 to be our estimate of 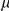, where 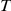 is the total number of mutants considered (doubles and singles). Then, the coefficient of determination is
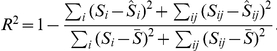
When 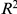 was close to 1, the model explained a large amount of the variation. For a formal test, we used an approach analogous to an 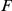 test. The sum of squared error is defined by
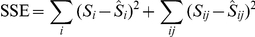
and the sum squared total is
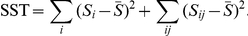
The sum of squares model is then the difference 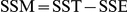. The degrees of freedom for SST is 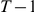, and the degrees of freedom for SSE is 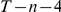, where 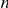 is the number of single mutants. The degrees of freedom for SSM is then 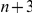. Therefore the 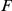 statistic would be
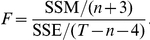
The standard 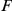 distribution may not hold because of the nonlinear nature of the model. All statistical analyses were done in R [50].

### Fitting the model to our data

To analyze our data, we shifted all fitnesses, which are given in units of doublings per hour, by subtracting a fitness value of 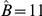 from each. This shifting allowed our model to address only fitnesses in the observed range without making predictions about the phenotype-fitness relationship for very low fitness values. Because of the simplicity of the model, it may not accurately describe the behavior far outside the range of our data. We could not shift by the wild-type fitness because two double mutants had fitnesses below that of the wild-type, which would have given negative fitness values. Therefore, we shifted by the largest integer value that was less than all observed fitnesses. The degrees of freedom for SSE becomes 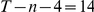, and the degrees of freedom for SSM becomes 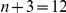. Note that the scale of the phenotypes is arbitrary, as a change in the phenotype scale can be absorbed by a change in the gamma scale parameter.

### Minimization algorithm

The minimization problem given by equation (2) is an 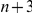 dimensional problem, where 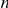 is the number of single mutations. We used the following algorithm to solve this problem.

1. Begin with an initial guess for 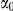, 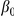, and 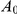.
2. For each fitness value 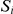 for the single mutants, solve for the two possible phenotypes 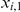 and 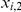 using 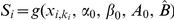, where 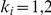. The two possible phenotypes for each single mutant represent the points of equal fitness on either side of the peak in the hypothesized phenotype-fitness map.
3. For each single mutation, a pair of possible phenotypes is denoted by the array
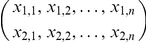
For each single mutant, choose one phenotype from each column to form a row of 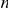 phenotypes. Denote the set of all row vectors by 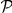. Among all arrays of phenotypes in 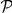, choose the one that minimizes the fitness effects of the doubles.
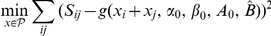

4. Denote the phenotypes solved for in steps 2 and 3 by 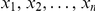. Fix the phenotypic values and minimize
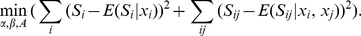
The solution can be used as input in step 1. The whole process can then be iterated until the solved values of 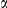, 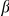, and 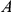 no longer change.

### Model comparisons

To compare the gamma model to the model of Martin et al. [40], we simulated the expected distributions of the deviations from additivity (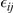 in our notation) under the two models. Parameter values were selected such that the two models yielded the same distributions for single beneficial mutations. For both models, we assumed the distribution of fitness effects followed the generalized Pareto distribution (GPD) with shape parameter 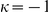 as estimated previously for the single mutations [47], [51]. The GPD with 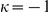 corresponds to a uniform distribution. We used the maximum observed fitness of the single mutations as our estimate for the upper bound and used the smallest observed fitness for a beneficial mutation as the lower bound.

To simulate 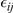
's under the gamma model, we chose nine fitness effects from the uniform distribution and mapped them to phenotypes using the inverse of the fitted gamma function. Each fitness value could be mapped to either side of the optimum; we selected the side at random. We assumed additivity of the phenotypes and generated the phenotypes of 18 double mutants. Double mutants were selected to match the pattern in our empirical data. Fitness was calculated for each on the basis of the gamma curve with normal error added from the estimated error. Deviations from addivity were calculated as described above. We generated 1,000 replicate data sets. This model requires imputation of nine phenotypes and estimation of four gamma parameters and the error parameter.

To simulate 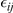
's under the model of Martin et al. [40], we noted that Fisher's geometrical model predicts a GPD distribution of beneficial fitness effects with 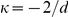, where 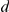 is the number of phenotypic dimensions [52]. Therefore, the number of dimensions for our data is 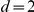. We used the same upper and lower bounds on fitness as for the gamma model and a two-dimensional geometrical model with a Gaussian phenotype-fitness map. The wild type was assumed to be one phenotypic unit from the optimum. Given phenotype values 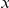 and 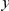, the fitness function is
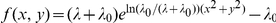
where 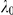 is the fitness of the wild type, and 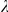 is the difference between the maximum fitness and the wild-type fitness. This form was selected to satisfy several constraints. We wanted 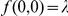 and, for simplicity, 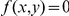 when 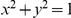. The final constraint shifted the floor of the function to 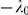; the location of this floor was not found to affect the results significantly. To generate our distribution of deviations from additivity, we simulated nine phenotypes at random within the circle defined by the fitness of the smallest-effect mutation, created 18 double mutants by vector addition, and mapped the single and double mutants to fitness to calculate the deviations from additivity. Double mutants were selected to match the pattern in our empirical data. We simulated 1,000 replicate data sets. This model requires the estimation of two parameters.

To compare the fit of the two models, we calculated AIC scores for each model, where 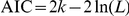. The number of parameters for the gamma model is 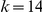 and 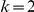 for the model of Martin et al. We approximated likelihoods (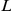) from the histogram densities.
